# Von Willebrand Factor as a Therapeutic Target in Thrombotic Disorders

**DOI:** 10.1055/a-2665-2313

**Published:** 2025-08-11

**Authors:** Yvonne K. Jongejan, Bart J.M. van Vlijmen, Jeroen C.J. Eikenboom

**Affiliations:** 1 Division of Thrombosis and Hemostasis, Department of Internal Medicine, Einthoven Laboratory for Vascular and Regenerative Medicine, Leiden University Medical Center, Leiden, The Netherlands

**Keywords:** von Willebrand factor, thrombosis, therapeutic agents

## Abstract

Von Willebrand factor (VWF) plays an important role in primary hemostasis. Dysregulated plasma VWF levels are implicated in various pathological conditions. Reduced or dysfunctional VWF is associated with bleeding, known as von Willebrand disease. Whereas elevated plasma VWF levels may give rise to an increased risk of developing arterial thrombotic events. In general, antithrombotic strategies in arterial thrombosis primarily focus on inhibiting platelet aggregation; however, treatment failure, antiplatelet drug resistance, and adverse bleeding tendencies underscore the necessity for the development of more efficacious and safer therapeutic modalities. Targeting VWF presents an interesting therapeutic approach as it operates independently of platelet activation pathways for platelet-rich thrombus formation. Over time, several VWF inhibitors have progressed to clinical application for thrombosis management, with ongoing research endeavors exploring novel compounds targeting VWF. This review provides a comprehensive overview of the evolution of VWF-targeting therapeutic agents, elucidating their current developmental stages, clinical indications, and evaluating their respective advantages and limitations.

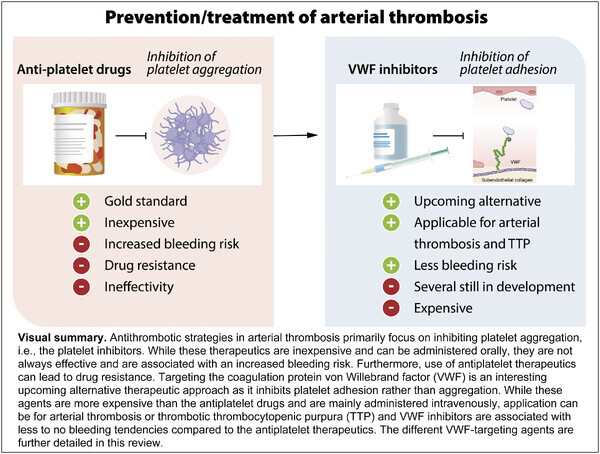

## Introduction

Von Willebrand factor (VWF), a vital multimeric plasma glycoprotein, is primarily synthesized in endothelial cells and megakaryocytes. It resides in storage organelles called Weibel–Palade bodies in endothelial cells and α-granules in platelets. 1 2 VWF plays a pivotal role in platelet-rich thrombus formation through interaction between its A1 domain and the platelet glycoprotein (GP)Ib receptor. 3 Upon vascular injury, VWF secretion is enhanced, and circulating VWF binds to exposed subendothelial collagen through its A3 domain, initiating platelet adhesion and aggregation. 1 3 4 Notably, VWF becomes active in regions characterized by high intravascular shear forces. This high shear stress is essential for the VWF protein to adopt the appropriate conformation to facilitate platelet adhesion; without high shear, VWF's thrombogenic potential diminishes. 5

The significant role of VWF in hemostasis was initially described through studies involving patients with von Willebrand disease (VWD). VWD, the most prevalent inherited bleeding disorder, is caused by either qualitative or quantitative defects, leading to reduced VWF activity. 6 Elevated plasma VWF levels, on the other hand, have been implicated in the pathophysiology of (arterial) thrombosis, which could lead to coronary heart disease, myocardial infarction, and ischemic stroke. 7 8 9 The potential causative role of high VWF levels is supported by findings indicating that VWD patients with low levels of VWF exhibit a reduced risk of developing arterial thrombotic events. 10 11 12 13

Current therapeutic strategies for preventing arterial thrombotic events predominantly focus on inhibiting platelet aggregation by targeting platelet activation pathways, either through acting as an antagonist for membrane receptors such as adenosine diphosphate (ADP) receptor P2Y12, Proteinase activated receptor 1 (PAR1), GPIIb/IIIa and GPIbα, or by modulating intracellular signaling pathways including Thromboxane A _2_ (TXA _2_ ) and cyclic nucleotides. 14 While aspirin, through irreversible inhibition of cyclooxygenase targeting the TXA _2_ pathway, and clopidogrel, by blocking the P2Y12 receptor's interaction with ADP, are regularly used to inhibit platelet aggregation, they are also associated with an increased risk of adverse bleeding. 5 14 15 Moreover, clopidogrel also exhibits other clinical limitations, including high inter-individual variability of the inhibitory effect in platelets and susceptibility to drug–drug interactions. 16 In addition, resistance to clopidogrel has been reported in numerous patients, and, albeit with a rare occurrence, there are also reports of resistance to aspirin. 17 18 Aspirin resistance is possibly associated with genetic variations (polymorphisms) in the COX-1 or COX-2 genes. 19 For clopidogrel, the presence of a certain polymorphism of the CYP2C19 gene has been shown to be important for the degree of clopidogrel metabolism. 20 Consequently, there remains an unmet medical need for the development of novel anti-thrombotic agents aimed at preventing (secondary) cardiovascular events with enhanced safety and efficacy.

**Fig. 1:**
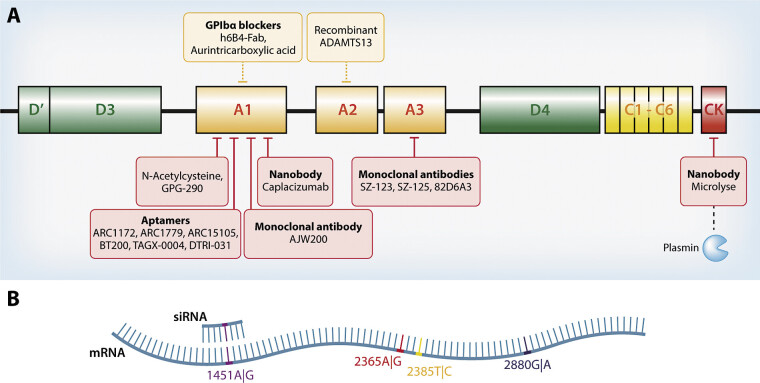
Overview of all therapeutic agents discussed and where they target von Willebrand factor (VWF). ( **A** ) Schematic view of the mature VWF protein. The boxes include the different classes discussed in the review, each box representing a class. Red boxes are direct inhibitors of the VWF function by binding to a certain region of the VWF protein. The yellow boxes include agents that indirectly inhibit the function of VWF. ( **B** ) Small interfering RNAs (siRNAs) targeting the VWF gene are specifically designed to silence only one allele of the VWF gene. This approach focuses on partial inhibition of VWF protein production, maintaining VWF function. Indicated in purple, red, yellow, and blue are the common genetic variants of interest on the VWF gene to be targeted using the siRNAs.

VWF has long been investigated as a therapeutic target, but recent advances have rejuvenated the field through the development of novel therapeutic agents. VWF is an interesting target as it acts independently of the platelet activation pathways for platelet-rich thrombus formation. 21 Instead, the interaction of VWF with platelet GPIbα facilitates a reversible binding of platelets to the subendothelial matrix. 21 22 VWF inhibitors can block its interaction with either GPIbα, leading to inhibition of the crosslinking of soluble VWF that is released into the circulation by shear-activated endothelium, or the subendothelial collagen, which inhibits the immobilization of VWF on the subendothelial matrix. 21 As VWF only becomes active in regions with high shear forces, VWF inhibitors would specifically act at these sites. This would lead to a relatively intact hemostasis in the venous circulation, where other mechanisms dominate. 23 This review delves into therapeutic agents targeting VWF, both clinically approved and in development, either through direct action on VWF itself or by targeting its ligands/receptors ( Fig. 1A ), and also addresses their advantages and limitations.

## Aptamers

Aptamers, a therapeutic class of RNA and DNA oligonucleotides, selectively target and bind molecules through specific, high-affinity interactions. 5 14 Aptamer identification and development rely on an *in vitro* selection methodology known as systematic evolution of ligands by exponential enrichment (SELEX). 2 23 24 Notable advantages of aptamers include their minimal immunogenicity and easy modification to improve bioavailability and pharmacokinetics. 14 The first aptamer-based therapeutic that reached clinical approval was later discontinued by the European Medicines Agency. 25 This particular aptamer, Pegaptanib (Macugen, Pfizer), targeted the vascular endothelial growth factor, serving as an inhibitor against macular degeneration. 26 Recently, a second aptamer, called Avacincaptad pegol (Izervay), was clinically approved to be used for geographic atrophy, an advanced stage of age-related macular degeneration. 27 This aptamer inhibits the cleavage of complement protein C5, thereby blocking the recruitment of the inflammasome and formation of the membrane attack complex. 28

**Fig. 2:**
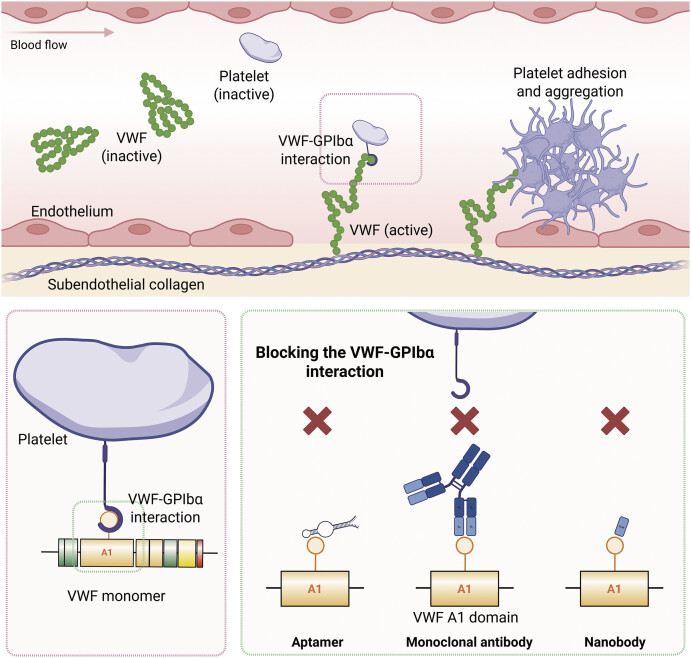
Mechanism of action of VWF A1 domain targeting therapeutic classes. The binding of von Willebrand factor (VWF) to platelets is essential for the initiation of platelet adhesion and aggregation. At the site of vascular damage, interaction with the subendothelial collagen activates VWF, resulting in a conformational change, which exposes the VWF A1 domain. The A1 domain specifically can bind to the platelet receptor glycoprotein 1b-α (GPIbα), facilitating the interaction between VWF and GPIbα. Aptamers, monoclonal antibodies, and nanobodies can interfere with this interaction by binding to the VWF A1 domain, thereby blocking the A1 domain from binding to GPIbα.

Aptamers designed to inhibit VWF target its A1 domain, disrupting the interaction between the A1 domain and GPIbα ( Fig. 2 ), thereby blocking VWF-mediated platelet adhesion, leading to potential prevention of pathological thrombotic events. 5 Aptamers act upstream in the cascade of platelet thrombogenesis, distinguishing their approach from traditional platelet inhibitors. Consequently, aptamers and platelet inhibitors have the potential to act complementary to each other, which could enhance their therapeutic effect. 22

### ARC1172 and ARC1779

ARC1172, a 41-mer DNA aptamer, exhibits a high binding affinity to the VWF A1 but is highly susceptible to nuclease-induced degradation. 23 To overcome this limitation, a modified version was designed, with improved resistance to nucleases but without compromising its binding affinity for the VWF A1 domain. This modified aptamer, known as ARC1779 or Egaptivon pegol, is a 40-mer aptamer appended with a 20-kDa polyethylene glycol (PEG) group. 4 14 23 ARC1779 was designed with the objective of preventing microthrombus formation, particularly in thrombotic thrombocytopenic purpura (TTP). 29 Additionally, it was also explored as a potential antithrombotic agent for acute coronary syndrome (ACS). 5 ARC1779 demonstrates a rapid onset of action, achieving its maximum inhibitory effect within 7 to 30 minutes, which compares favorably to clopidogrel, which requires 2 hours to reach its maximum inhibitory effect. 5 30 ARC1779's specificity toward the binding of VWF to platelets minimizes bleeding risk, as demonstrated in healthy volunteers, and it can be reversibly neutralized using complementary antidotes based on its sequence. 5 However, ARC1779 has a short half-life of approximately 2 to 3 hours, necessitating continuous administration. 4 31 32 In a phase IIa trial (ARC1779–004), the safety and clinical feasibility of ARC1779 was assessed in acute TTP patients. 33 The compound was tolerated well and showed comparable pharmacokinetic and pharmacodynamic profiles consistent with those observed in the prior phase I trial involving healthy volunteers. 4 33 Additional phase II clinical trials with ARC1779 for acute TTP and carotid endarterectomy were initiated; however, both were prematurely terminated due to lack of funding. 29 34 Since then, no further clinical trials on ARC1779 have been conducted (for an overview of the developmental phase of the therapeutic agents, see Table 1 ). Nonetheless, knowledge gained from ARC1779 paved the way for the development of a new generation of chemically modified aptamers targeting VWF.

**Table 1:** Overview of therapeutic agents targeting VWF, including their mechanism of action and current developmental stage

Name the therapeutic agent	Mechanism of action	Developmental stage
Aptamers		
ARC1172	Inhibits VWF-GPIb interaction via binding the A1 domain in VWF	Stopped during preclinical development
ARC1779	Inhibits VWF-GPIb interaction via binding the A1 domain in VWF	Early termination during the phase II clinical trial
ARC15105	Inhibits VWF-GPIb interaction via binding the A1 domain in VWF	Stopped during preclinical development
BT200	Inhibits VWF-GPIb interaction via binding the A1 domain in VWF	Development stopped for secondary stroke prevention after phase I clinical trial; completed phase IIa clinical trial for hemophilia A and von Willebrand disease
TAGX-0004	Inhibits VWF-GPIb interaction via binding the A1 domain in VWF	Preclinical development
DTRI-031	Inhibits VWF-GPIb interaction via binding the A1 domain in VWF	Phase II clinical trial planned
Monoclonal antibodies		
AJW200	Inhibits VWF-GPIb interaction via binding the A1 domain in VWF	Development stopped after phase I clinical trial
82D6A3	Inhibits the binding of VWF to collagen by binding the A3 domain in VWF	Preclinical development a
SZ-123	Inhibits the binding of VWF to collagen by binding the A3 domain in VWF	Preclinical development
SZ-125	Inhibits the binding of VWF to collagen by binding the A3 domain in VWF	Preclinical development a
Nanobodies		
Caplacizumab (Cablivi)	Inhibits VWF-GPIb interaction via the VWF A1 domain	Clinically approved in 2019
Microlyse	Facilitates the binding of plasminogen to VWF to trigger degradation of platelet-VWF complexes by plasmin	Preclinical development
Modulators of UL VWF multimers		
Recombinant ADAMTS13 (Adzynma)	Increases ADAMTS13 activity to cleave UL VWF multimers	Clinically approved in 2023
N-acetylcysteine	Reduces UL VWF multimer size	Clinical development
Other VWF inhibitors		
GPG-290	Inhibits VWF-GPIb interaction via binding the A1 domain in VWF	Preclinical development a
siRNAs	Inhibits VWF protein translation	Preclinical development
Platelet receptor blockers		
h6B4-Fab	Inhibits VWF-GPIb interaction by blocking the GPIb receptor	Preclinical development a
Aurintricarboxylic acid	Inhibits VWF-GPIb interaction by blocking the GPIb receptor	Preclinical development a

Abbreviations: ADAMTS13, a disintegrin and metalloproteinase with a thrombospondin type 1 motif, member 13; GPIb, glycoprotein Ib; UL VWF, ultra-large VWF; VWF, von Willebrand factor.

a No updates on therapeutic development for at least 10 years.

### ARC15105

One next-generation aptamer is the RNA aptamer ARC15105, which was chemically modified with a 40-kDa PEG moiety and fully 2'O-Methyl (2'-OME) chemistry to enhance stability. Developed as a potential therapeutic for myocardial infarction prevention, 22 31 ARC15105 exhibits a similar high degree of specificity for the VWF A1 domain. In studies using porcine aortas, ARC15105's inhibitory effect exceeded that of ARC1779 twofold, with a significantly prolonged duration of effect and a high bioavailability of 98%. 22 31 The addition of the 40-kDa PEG group substantially prolonged ARC15105's half-life to approximately 65 hours. 4 31 However, ARC15105 lacks thermostability due to its stem-loop structure, leading to structural instability at elevated temperatures of 37°C and above. To improve stability, four extra base pairs and a free amine group at the 5′-end were added, which resulted in a more potent and stable aptamer known as BT100. While BT100 showed improved stability and potency, it was not appended with PEG, shortening its half-life. A 40-kDa PEGylated version, BT200, was subsequently created. 22

### BT200

BT200, also known as Rondoraptivon pegol and Rondaptivon pegol, was initially developed for secondary stroke prevention. 22 32 35 36 37 Like ARC15105, BT200 is a pegylated RNA aptamer that can bind with high affinity and specificity to the VWF A1 domain, blocking the interaction between VWF and GPIbα. 35 Preclinical studies with cynomolgus monkeys demonstrated an 80% reduction in VWF activity and a limited 19% reduction in VWF antigen (VWF:Ag) levels, supporting the hypothesis that lowering VWF activity to 20% could reduce secondary stroke risk. 22 32 37 However, this level of VWF suppression poses bleeding risks. To mitigate this, a reversal agent, BT101, was developed and demonstrated in cynomolgus monkeys to rapidly reverse BT200's inhibitory effects. 35 BT200 possesses an extended half-life of up to 103 hours, which is notably longer than its predecessors ARC15105 (approximately 65 hours) and ARC1779 (2–3 hours), 22 and allows for potential weekly therapeutic administration. 37 However, when BT200 was tested in a first-in-human clinical trial involving healthy volunteers, it exhibited an intriguing phenomenon: while VWF activity levels were decreased upon BT200 treatment, similarly to the preclinical studies, VWF:Ag and plasma coagulation factor VIII (FVIII) activity were increased. These findings, combined with the development of venous thrombosis and thrombophlebitis at the sites of venous catheter insertion, raised concerns about the potential for heightened hemostatic activity upon BT200 treatment, rendering it less suitable as a therapeutic approach for secondary stroke prevention. 38 Consequently, the potential clinical application of BT200 as a pro-hemostatic agent for treating hereditary bleeding disorders, including VWD type 2B and hemophilia A, was investigated in a phase IIa clinical trial. 39 40 In VWD type 2B patients with thrombocytopenia, treatment with BT200 led to improved platelet counts, VWF multimer patterns, VWF:Ag, and FVIII levels. 39 Similarly, in patients with nonsevere hemophilia A, both mild and moderate, BT200 treatment led to increased FVIII and VWF:Ag levels. 40 However, due to the intrinsic inhibition of the VWF A1 domain, the ratio of VWF activity (VWF:RCo) to VWF:Ag was reduced. While these changes suggest a physiological impact, the overall effect on the clinical phenotype of both VWD type 2B and hemophilia A remains to be fully understood. Notably, the prolonged half-life of VWF observed in this context can be attributed to the biological mechanism of BT200, which differs from that of other described VWF A1 domain inhibitors. BT200 specifically interacts with the A1 domain in proximity to four conserved lysine residues (K1405, K1406, K1407, and K1408), which attenuates VWF binding to macrophage lipoprotein receptor-related protein 1 clusters II and IV and subsequently reduces macrophage-mediated clearance of VWF. 41 Beyond its direct effect, BT200 also demonstrated the ability to extend the half-life of FVIII substitution products when used in combination with one of these products, offering the potential to reduce the frequency of FVIII substitution administrations. 40 Despite the limited sample sizes in this phase IIa trial, the results were encouraging, paving the way for future phase IIb/III trials targeting VWD type 2B or hemophilia A.

### TAGX-0004

TAGX-0004 is a DNA aptamer specifically targeted toward the VWF A1 domain. 42 Its structure, containing the artificial hydrophobic base 7-(2-thienyl)imidazo[4,5-b]pyridine (Ds) and a mini-hairpin, enhances binding affinity and resistance to degradation by nucleases. 43 An *in vitro* comparison between TAGX-0004, ARC-1779, and the anti-human VWF nanobody caplacizumab demonstrated that TAGX-0004 was a 10-fold more potent inhibitor of the VWF A1 domain and of VWF function when contrasted with ARC1779, and exhibited comparable potency to caplacizumab. The heightened inhibitory activity of TAGX-0004 compared with ARC1779 was even more pronounced at the assessment of platelet thrombus formation under high shear stress conditions, as TAGX-0004 proved to be at least 20 times more potent than ARC1779. Furthermore, it offers an added advantage over caplacizumab, as antidotes can be prepared using neutralizing agents complementary to the aptamer. However, it is essential to acknowledge certain drawbacks associated with TAGX-0004, such as high costs and the potential for adverse bleeding events. When developed further, TAGX-0004 has the potential to be applied for acquired TTP (aTTP), ACS, and cerebral infarction. 42 Nevertheless, further (pre)clinical development has not been reported to date.

### DTRI-031

DTRI-031, also known as BB-031, originates from the 2'Fluoro-pyrimidine-modified RNA aptamer Ch-9.14-T10. 44 Clone R9.14, together with a rapid and effective complementary antidote, was selected for its high binding affinity to VWF and its potential to inhibit platelet aggregation. 45 To facilitate large-scale synthesis, R9.14 was truncated from 80 to 60 nucleotides by deleting nucleotides from the 3′-end, and underwent a cholesterol modification at the 5′-end, giving rise to Ch-9.14-T10. This compound retained the potency of R9.14 while boasting a prolonged circulating half-life *in vivo* as well as being a robust thrombosis inhibitor, albeit with an increased risk of significant blood loss at sites of vascular or tissue damage. To counteract adverse bleeding, an antidote was designed to rapidly reverse the activity of Ch-9.14-T10. 46 Although Ch-9.14-T10, with its 60-nucleotide length, was already smaller than the original R9.14, for large-scale synthesis, especially for use in large animal models and for clinical application, an even smaller aptamer was required.

Truncation of Ch-9.14-T10 led to the development of DTRI-031, an aptamer with a length of 35 nucleotides, a 5-nucleotide uracil tail at the 3′-end, and additional chemical modifications for improved nuclease resistance. In experimental settings with human whole-blood samples, DTRI-031 demonstrated its potency by dose-dependently inhibiting platelet adhesion and aggregation. In murine models with vessel injury, treatment with DTRI-031 resulted in patent vessels, in contrast to mice subjected to less or no aptamer treatment as they developed vessel occlusion. The potential of DTRI-031 as a therapeutic extends beyond thrombosis prevention, as it was also able to restore blood flow in murine arterial thrombosis and canine carotid occlusion models. Furthermore, DTRI-031 outperformed recombinant tissue plasminogen with its ability to recanalize vessels. 44 Additionally, DTRI-031 displayed its thrombolytic potential by inducing thrombolysis in an *in vitro* microfluidic model of reperfusion of a nearly occlusive arterial thrombus. 47 BB-025, a 16-nucleotide complementary antidote to DTRI-031, demonstrated the ability to control hemorrhagic bleeding in murine and canine models. 44 48 The subsequent phase I clinical trial in healthy volunteers showed that DTRI-031 was well-tolerated, bleeding events were infrequent, and VWF binding and *ex vivo* clot formation were dose-dependently inhibited. 49 These promising outcomes have set the stage for a phase II clinical proof of concept trial, intended to assess DTRI-031's potential in patients suffering from acute ischemic stroke. Preparations for this trial are currently underway.

## Monoclonal Antibodies

Monoclonal antibodies (mAbs) are a class of therapeutic antibodies. They are created using the hybridoma technique in which immunized cells derived from a donor organism are fused with myeloma cells, giving rise to a hybrid cell line known as a hybridoma, which has high replicative capacity and the capability to produce antibodies. 50 Numerous mAbs designed to inhibit the function of VWF have been investigated to assess their therapeutic potential. mAbs that have been used to investigate the mechanism and function of VWF 51 52 53 will not be discussed in this review.

### AJvW-2 and AJW200

AJvW-2 is a murine mAb that interacts with an epitope that is present in the VWF A1 domain of humans and other animal species, including the pig, rabbit, dog, guinea pig, and rat. AJvW-2 interferes with the binding of VWF to GPIbα, preventing platelet adhesion under high shear forces without increasing the bleeding risk ( Fig. 2 ). 54 55 56 The interaction of AJvW-2 with VWF is highly specific, as platelet aggregation, mediated by other factors like ADP, collagen, U46619, and low shear stress, was not inhibited. 56 Initially designed for use after coronary interventions to prevent new thrombotic events in the coronary arteries and inhibit restenosis, 54 AJvW-2 demonstrated anti-thrombotic potential in various animal models, but was not suitable for clinical application due to the potential immunogenicity of its murine immunoglobulin (IgG) _1_ . Consequently, a humanized version of this mAb, AJW200, was developed. 57

Like its precursor AJvW-2, AJW200 was derived from mouse myeloma cells. However, by grafting the mouse-determining regions onto a human IgG _4_ framework, immunogenicity was reduced. Similar to its murine counterpart, AJW200 specifically inhibited platelet adhesion under high shear stress in cynomolgus monkeys and dogs. This was, in contrast to treatment with an antibody targeting platelet GPIIb/IIIa, without extensively prolonging bleeding times. 57 58 In a phase I clinical trial with 24 healthy male volunteers, intravenous infusion of AJW200 showed a dose-dependent inhibitory effect in the ristocetin cofactor activity, along with prolonged closure times in the platelet function analysis, which lasted up to 12 hours at the highest dose. However, no significant changes were observed in VWF:Ag levels, VWF multimer pattern, VWF cleaving protease activity, or in FVIII activity. There were no serious adverse events or signs of immunogenicity recorded, and skin bleeding time was not prolonged. 59 Despite the promising outcomes, further follow-up trials with AJW200 have not been reported.

### 82D6A3

82D6A3 is a mAb that specifically targets the A3 domain of VWF and effectively blocks its binding to type I and III fibrillar collagens. Initially investigated to assess the mechanism of VWF, 60 61 its antithrombotic potential was first described when tested in a modified Folts model in baboons. 62 The Folts model can be used to study platelet aggregation, thrombus formation, and embolization under conditions of stenosed and damaged arteries. A parameter for occlusive thrombosis development measured by this model is the reduction in coronary flow, which is called cyclic flow reduction. 63 In the baboon model investigating 82D6A3′s antithrombotic potential, when compared with a GPIIb/IIIa-targeting mAb, significantly reduced cyclic flow reductions were observed, and the bleeding time was not affected. 62 This effect is probably attributed to the mechanism of 82D6A3, which primarily affects platelet-dependent hemostasis under high shear conditions. To advance to clinical applicability, 82D6A3 was humanized to reduce antigenicity while maintaining affinity. Similar to the humanization process of AJW200, 82D6A3 was humanized by grafting the murine single-chain variable fragment onto a human IgG _4_ . 64 Humanized 82D6A3 showed promise in preventing restenosis in a baboon model for coronary artery in-stent stenosis, hinting at potential application in patients with in-stent stenosis. 65 While the humanized 82D6A3 was constructed with the intention of further preclinical and clinical development, 64 no updates on progress or outcomes have been published since.

### SZ-123 and SZ-125

SZ-123 and SZ-125 are murine antibodies, designed to specifically target the VWF A3 domain. 66 Similar to 82D6A3, these antibodies block the binding of VWF to collagen type III and impede the VWF-dependent platelet adhesion to exposed collagen under high shear stress conditions. 66 67 SZ-123 and SZ-125 differ from 82D6A3 as they can also inhibit platelet aggregation induced by ristocetin, botrocetin, or bovine plasma. 66 Interestingly, despite the functional similarities between SZ-123 and SZ-125, they recognize different types of epitopes, with SZ-125 identifying a linear epitope resistant to proteolysis, while SZ-123 recognizes a more complex, discontinuous epitope, requiring a native confirmation for VWF recognition. 68 SZ-123 was further explored *in vivo* in Rhesus monkeys, demonstrating antithrombotic effects in an arterial platelet thrombosis model. 67 While the interaction of VWF to collagen was effectively blocked, SZ-123 did not affect VWF:Ag levels, platelet count, bleeding times, or coagulation parameters. 67 To reduce immunogenicity, a mouse/human chimeric variant of SZ-123, named MHCSZ-123, was constructed. Similar to its murine counterpart, MHCSZ-123 effectively inhibited the binding of VWF to collagen type III and arterial platelet thrombosis in Rhesus monkeys. 69 Despite the authors expressing an interest in validating this antibody in clinical trials to prevent acute arterial thrombotic syndromes, no further progress has been reported.

## Nanobodies

Nanobodies represent a special class of monoclonal antibodies, as they consist solely of a functional heavy chain. These heavy-chain antibodies exhibit smaller dimensions compared with a full antibody, primarily due to the absence of light chains and the first constant C _H_ 1 domain within the heavy chain. 70 They are based on the smallest functional fragments of single-chain antibodies that occur naturally in members of the Camelidae family. Nanobodies offer several notable advantages, including their adaptability to various formats and their capacity to combine the benefits associated with full antibodies and small molecule drugs, a characteristic facilitated by their small size. 71

### Caplacizumab

Caplacizumab, also known as Cablivi, ALX-0081 or ALX-0681, is a bivalently humanized nanobody designed to target the VWF A1 domain, inhibiting its interaction with GPIbα ( Fig. 2 ). 72 73 74 Initially, preclinical 72 and clinical phase I 75 76 and II trials investigated caplacizumab's potential for thrombosis prevention in high-risk ACS patients undergoing percutaneous coronary interventions (PCI). 72 77 Caplacizumab was explored as an alternative to abciximab, which was the standard recommendation for PCI procedures at the time of caplacizumab's development, but was also associated with increased bleeding complications and costliness. 78 While preclinical and phase I trials demonstrated safety, the phase II trial in ACS patients failed to meet the primary endpoint of reducing bleeding events by 40% compared with abciximab treatment. Consequently, the focus shifted to the potential application of caplacizumab in aTTP patients. 79 Three phase I studies explored administration routes, including single or multiple intravenous injections, intravenous infusions, and subcutaneous injections, demonstrating tolerability without significant bleeding or serious complications. 73 The subsequent phase II TITAN trial investigated the safety and efficacy of caplacizumab as an adjuvant therapy for plasma exchange therapy in aTTP patients. 73 74 Although aimed at a sample size of 110 patients, recruitment was halted prematurely at 75 patients due to persistent recruitment challenges. The included 75 aTTP patients either received caplacizumab or a placebo in addition to the standard-of-care treatment, which included daily plasma exchange and immunosuppressive therapy. The primary endpoint was the time to normalization of the platelet count, which was significantly reduced in the caplacizumab group. Other observations in the caplacizumab group included no deaths, a smaller number of exacerbations but also more relapses in patients with a severe ADAMTS13 (a disintegrin and metalloproteinase with a thrombospondin type 1 motif, member 13) deficiency within 10 days after stopping the treatment, and although being considered mild or moderate, an increased number of bleeding events. 74 The outcomes of the TITAN trial led to the phase III HERCULES trial, which aimed to test the safety and efficacy of caplacizumab in aTTP patients in addition to the standard-of-care treatment. Treatment with caplacizumab resulted in a shorter time to platelet count normalization, total plasma exchange treatment duration, intensive care unit stay, overall hospitalization duration, and a lower volume of exchanged plasma. Similar to the other studies with caplacizumab, the HERCULES trial also demonstrated an increased risk of bleeding events as one of the main adverse effects, with 65% of the patients in the caplacizumab group suffering from adverse bleeding, compared with 48% of the patients in the placebo group. To prevent relapses as in the TITAN trial, the HERCULES trial allowed extended caplacizumab treatment, resulting in fewer relapses. 80 Despite increased bleeding risks, the added value of caplacizumab ultimately led to clinical approval of caplacizumab use alongside standard-of-care treatments in aTTP patients. Since the application of caplacizumab in the clinic, mortality rates for TTP have dropped to 0 to 6% versus mortality rates of 10 to 20% before the introduction of caplacizumab. However, controversies persist regarding high costs, clinical benefits, and adverse effects, requiring individualized treatment regimens. Ongoing clinical trials are being performed to show caplacizumab's value in terms of reducing mortality, disease burden, and (long-term) complications in TTP patients. 81

### Microlyse

Microlyse, also known as TGD001, is a therapeutic agent acting as a thrombolytic agent. It is a fusion polypeptide, including a nanobody designed to target the C-terminal CT/CK domain of VWF and the protease domain of urokinase plasminogen activator (uPA). 82 83 Microlyse is being developed for use in acute TTP attacks, particularly in cases of aTTP, where recombinant ADAMTS13 (rADAMTS13) may not be effective due to the presence of autoantibodies against ADAMTS13. 83 The strategy of Microlyse is to replace the VWF cleaving properties of ADAMTS13 with plasmin, which has been demonstrated to efficiently cleave VWF strings when administered in therapeutic dosages. 84 To prevent an increased bleeding risk associated with systemic uPA administration, the catalytic domain of uPA was fused with a VWF-targeting nanobody, which should restrict plasminogen activation solely to the surface of VWF. *In vitro* comparisons of Microlyse, caplacizumab, tissue plasminogen activator (tPA), and rADAMTS13 on the ability to disrupt platelet-VWF complexes revealed that Microlyse was able to disrupt these complexes with comparable efficiency to caplacizumab and rADAMTS13, whereas tPA treatment was less effective. The addition of the fibrinolysis inhibitor tranexamic acid to Microlyse, however, prevented disruption of the platelet-VWF complexes. In *Adamts13 ^−/−^* -deficient mice, which are a representative *in vivo* TTP model, Microlyse decreased thrombocytopenia. 82 Following the promising findings of Microlyse in the preclinical TTP models, an in vivo study was performed using acute ischemic stroke models. In this study, Microlyse was compared with the recombinant human tPA (alteplase), the clinically approved thrombolytic treatment for acute ischemic stroke. In comparison to *ADAMTS13* treatment, which is limited to VWF-dependent platelet-rich thrombi, is alteplase only applicable as treatment for fibrin-rich thrombi. Microlyse treatment, on the other hand, was hypothesized to have thrombolytic effects in both fibrin- and platelet-rich thrombi. Although the thrombolytic properties of Microlyse were similar to those of alteplase in the fibrin-rich acute ischemic stroke mouse model, in the platelet-rich acute ischemic stroke model, treatment with Microlyse led only to decreased lesion volumes and not increased reperfusion. 85 These findings indicated that besides its potential as a therapeutic for TTP, Microlyse also shows potential as a thrombolytic treatment for acute ischemic stroke. However, clinical trials are needed for validation, and a phase 1a trial assessing safety and tolerability in healthy volunteers is currently being conducted.

## Modulators of Ultra-Large VWF Multimers

Ultra-large VWF (UL VWF) multimers, the most thrombogenic forms of VWF, are predominantly located in Weibel–Palade bodies or α-granules and are only released at the site of vascular injury. 86 UL VWF multimers are more adhesive than smaller-sized multimers, which can lead to spontaneous binding to platelets even in the absence of vascular damage. 87 88 To regulate the thrombogenic properties of these UL VWF multimers, the protease ADAMTS13 cleaves these multimers at the VWF A2 domain, transforming them into smaller-sized multimers. 88 In case of decreased or deficient ADAMTS13, cleavage of UL VWF multimers is not feasible, potentially leading to the formation of microthrombi and subsequent TTP. 88 89 Therapeutic modulators of UL VWF multimers replace the function of ADAMTS13, facilitating the cleavage or disruption of UL VWF multimers into smaller-sized, less thrombogenic multimers.

### Recombinant ADAMTS13

rADAMTS13, also known as Adzynma, apadamtase alfa, TAK-755, BAX 930, and SHP655, is a recently approved therapeutic compound for hereditary or congenital TTP (cTTP). 90 91 In cTTP patients, mutations in the ADAMTS13 gene lead to a severe ADAMTS13 deficiency. 92 The standard-of-care treatment for acute TTP events in cTTP patients involves infusion with fresh frozen plasma, but not all patients benefit from this treatment due to plasma-related complications, such as allergic reactions, infections, or volume overload. 93 rADAMTS13 was initially only investigated as a therapeutic for cTTP and not for aTTP patients, whose ADAMTS13 deficiency is mediated by acquired inhibitory or noninhibitory auto-antibodies against ADAMTS13. 89 rADAMTS13 was first developed in several transient and stable cell lines. 92 94 To explore the potential of rADAMTS13 as a (prophylactic) treatment for cTTP patients, a phase I trial with 15 cTTP patients divided into three dose-cohorts assessed safety following rADAMTS13 treatment. All patients tolerated the drug well with no reported serious adverse events, and none of the patients developed anti-ADAMTS13 antibodies. However, because patients only received two rADAMTS13 dosages, long-term effects were yet to be studied in a subsequent phase III trial. 95 The phase III trials leading to clinical approval consisted of a pivotal study and a continuation study. 96 97 98 In the phase IIIa pivotal study, severe cTTP patients received either prophylactic rADAMTS13 or the standard-of-care treatment for 6 months, then crossed over to the alternate treatment for an additional 6 months. For the remaining 6 months, all patients received rADAMTS13 treatment. 96 97 During rADAMTS13 treatment, no acute TTP attacks occurred, in contrast to one event during standard-of-care treatment. 96 Plasma ADAMTS13 activity levels were consistently higher during rADAMTS13 treatment, and even with prolonged rADAMTS13 prophylaxis, none of the patients developed ADAMTS13 neutralizing antibodies, consistent with observations in the phase I trial. 95 96 The phase IIIb continuation study included 29 out of the 36 patients from the pivotal study, which formed the rollover cohort, and they continued rADAMTS13 treatment. In the continuation study, long-term safety and efficacy were evaluated. Similar to the pivotal study, no anti-ADAMTS13 antibodies developed, there were no reports of treatment-related adverse events, and no acute TTP attacks occurred during rADAMTS13 prophylaxis. 98 Following these phase III trials, rADAMTS13 received approval for use as a prophylactic and acute symptoms treatment in severe cTTP patients. 90 Phase II clinical trials evaluating rADAMTS13 in aTTP patients are ongoing. Additionally, rADAMTS13 has been explored for other indications, with a completed phase I clinical trial for sickle cell disease. 91

### N-Acetylcysteine

N-acetylcysteine (NAC), the acetylated variant of the amino acid L-cysteine, is a precursor of reduced glutathione and is indicated as a mucolytic agent to clear the airway from mucus in congestive and obstructive lung disorders. The main protein components of mucus are polymeric mucins, which are connected via disulfide bonds. NAC contains a free thiol compound that breaks these disulfide bonds, reducing mucin size. 99 Polymeric mucins have a similar structure to VWF multimers, suggesting that NAC can also potentially reduce the size and reactivity of the thrombogenic UL VWF multimers by breaking its disulfide bond. 99 100 The feasibility of NAC to reduce UL VWF multimers was demonstrated *in vitro* under static conditions and *in vivo* in *Adamts13 ^−/−^* mice. 99 NAC treatment in mouse and baboon models for aTTP also resulted in reduced UL VWF multimers but not in recovery of preexisting TTP symptoms. When administered prophylactically, however, NAC treatment prevented the onset of severe TTP symptoms. 101 In ischemic stroke models, NAC could not prevent occlusive thrombus formation, but the formed thrombi were unstable. Combining NAC with a GPIIb/IIIa inhibitor resulted in a synergistic effect on restoring arterial patency, thrombolytic efficiency, and subsequent improvement of ischemic stroke outcome. 102 Based on these varying results, DiNAC, the disulfide dimer of NAC, was investigated as a potentially more effective thrombolytic agent, demonstrating in an *in vitro* setting for arterial thrombosis complete lysis of platelet- and VWF-rich thrombi 103 but further studies on DiNAC have not been reported. NAC, however, was further evaluated clinically as a treatment for TTP. In contrast to the preclinical studies, where NAC was mostly used as a monotherapy, in patients, NAC is administered as an adjuvant therapy in combination with plasma exchange, glucocorticoids, and sometimes antibiotics. In the available case reports, a rapid platelet response and minimal adverse reactions were noted. 104 105 106 107 However, some patients experienced TTP exacerbations during NAC treatment, prompting suggestions that the treatment regimen needed adjustments in terms of dosing and duration. Additionally, it should be noted that high doses of intravenously administered NAC increase the risk of anaphylactic reactions. 108 Despite this, NAC is considered a widely available, low-cost, and safe adjuvant therapy for TTP. 107 A phase II pilot trial assessed NAC as an adjuvant thrombolytic agent to alteplase treatment in acute ischemic stroke, demonstrating limited differences at 3 months in terms of mortality, recanalization, overall good outcomes, or adverse effects between a combination of NAC and alteplase or alteplase alone. However, combined NAC and alteplase treatment led to an improved early neurological outcome, although larger trials should evaluate NAC's safety and efficacy as a thrombolytic agent. 109 NAC has also been investigated for thrombosis prevention, demonstrating prolonged occlusion times in an *in vitro* microfluidic model for arterial thrombosis and *in vivo* no occlusive thrombus formation in a modified Folts mouse model, although repeated high dosages led to hemorrhages at the site of injury. Further testing with NAC as a thromboprotective agent was suggested to explore effective lower maintenance doses to prevent bleeding events. 100

## Other VWF Inhibitors

Some other molecules, such as recombinant proteins, are also able to target and inhibit the VWF protein. These compounds have been studied for their anti-thrombotic potential. Additionally, small interfering RNAs (siRNAs), inhibiting the production of VWF, have been investigated for their anti-thrombotic potential.

### GPG-290

GPG-290 is a recombinant chimeric protein comprised of the N-terminal 290 amino acids derived from the human GPIbα subunit and linked to human IgG1Fc. 110 111 It incorporates two substitutions, consistent with naturally occurring gain-of-function mutations observed in VWF, exhibiting a 14-fold increase in binding affinity to VWF compared with platelet GPIbα, effectively blocking their interaction and subsequently inhibiting platelet adhesion. 110 Preclinical studies in canine models for coronary thrombosis showed dose-dependent prevention of thrombosis development, with only significant prolonged bleeding times at the highest dose. GPG-290 did not affect plasma VWF:Ag levels, VWF collagen binding, prothrombin time, or activated partial thromboplastin time. 110 111 In combination with clopidogrel, a lower dose of GPG-290 proved effective in improving coronary blood flow and antithrombotic potential in a coronary thrombosis model without adversely affecting bleeding times. 110 In cases of GPG-290-related prolonged bleeding, desmopressin infusion normalized bleeding times without compromising coronary blood flow. 111 Additional testing of GPG-290 in an in vivo mouse model for deep vein thrombosis demonstrated robust protection against venous thrombosis development. 112 Despite these promising results, GPG-290's clinical applications require further investigation.

### Small Interfering RNAs

In contrast to the therapeutic agents described above, which directly or indirectly inhibit the VWF protein or its function, small interfering RNAs (siRNAs) inhibit the expression of the *VWF* gene. siRNAs are short synthetic double-stranded RNA sequences, typically 19–23 nucleotides long, comprising a guide strand and an antisense strand. The complementarity of the anti-sense strand to the target mRNA leads to mRNA degradation, thereby preventing translation into protein. 113 Instead of blocking the function of the VWF protein, siRNAs therefore inhibit the production of the VWF protein, resulting in lower circulating but still functionally active VWF. Potent siRNAs can fully silence a gene, which could result in marginal remaining levels of VWF, which are associated with increased bleeding risk, as observed in VWD patients. 6 Increased bleeding risk due to treatment is also a major limitation of the other VWF-targeting agents discussed in this review. To mitigate bleeding risk while preventing thrombotic events, controlling the dose of siRNAs could limit knockdown to an effective but safe window of VWF levels, but this approach would require frequent monitoring to avoid too low VWF levels. Titration of siRNA dose is also complex, considering the wide range of VWF levels in patients and the intra-individual variation over time. An alternative approach involves allele-selective silencing of the *VWF* gene. In that strategy, siRNAs target a genetic difference between the two alleles of the VWF gene, silencing only one allele and leading to a maximal knockdown of around 50%, which is not expected to induce bleeding. In the context of VWD, *in vitro* and *ex vivo* proof-of-principle studies were conducted to demonstrate the potential of allele-selective silencing of the mutant allele in ameliorating disease phenotype. 114 115 As VWD can be caused by hundreds of different mutations, 116 instead of designing siRNAs specifically targeted toward every individual mutation, siRNAs were designed against one of the four most common single-nucleotide polymorphisms (SNPs) in the *VWF* gene of the Caucasian population ( Fig. 1B ). 114 117 This SNP-based allele-selective targeting of VWF is also an option for the indication of antithrombotic treatment. For thrombosis prevention, proof-of-concept studies using siRNAs targeting strain-selective murine *Vwf* were performed to assess the in vivo potential of allele-selective silencing of *Vwf* . 118 In crossbred heterozygous mice, the feasibility of allele-selective inhibition of *Vwf* was demonstrated, resulting in a knockdown of approximately 50%, which led to decreased thrombus development in the ferric chloride-induced thrombosis challenge in the mesenteric arterioles while retaining normal bleeding times upon a tail-clip bleeding challenge, suggesting that allele-selective siRNA-mediated silencing of *Vwf* is an effective and safe approach. 119 Preclinical studies using allele-selective siRNAs targeted toward the human *VWF* gene are yet to be performed.

## Indirect Inhibitors of VWF

Another class of inhibitors does not target VWF itself but rather blocks its binding to the platelet receptor GPIbα. Blocking of the platelet receptor hampers platelet adhesion and subsequently thrombus formation. 120

### Hour6B4-Fab and Aurintricarboxylic Acid

6B4 is a mAb and an inhibitor of platelet receptor GPIbα. While initially 6B4 treatment in baboons led to effective hindering of the binding of VWF to GPIbα, it also coincided with immediate and severe thrombocytopenia. 121 Using only the Fab fragments prevented thrombocytopenia, while the antithrombotic effects were powerful and with minimal to no prolongation of the bleeding time in a modified Folts model in baboons. 122 Yet, it was only effective before the initiation of thrombus development, unable to suppress further thrombosis development. 121 Subsequent development of a fully recombinant and humanized version with similar affinity, potency, and anti-thrombotic capacity to its murine parent was named h6B4-Fab. Despite mentioning the necessity for clinical trials to validate h6B4-Fab's therapeutic potential for acute thrombotic syndromes, 123 no clinical outcomes have been reported to date.

Aurintricarboxylic acid (ATA) is a triphenylmethyl dye compound, 124 and like h6B4-Fab, an inhibitor of platelet adhesion through interference of the binding of VWF to GPIbα. 125 Fractionating commercial ATA into ATA polymers led to a polymer size-dependent increase in inhibitory potency and selectivity to VWF, resulting in vivo in antithrombotic effects, improved patency, and prevention of rethrombosis in rat, canine, and hamster arterial thrombosis models but also increased bleeding times. 126 127 128 ATA also possesses the ability to inhibit thrombin-induced platelet aggregation. 127 Despite no further publications on ATA as a VWF inhibitor, numerous papers have reported on other biological properties of ATA. These studies, in combination with the reports on increased bleeding risk, suggest the likelihood of multiple pharmacokinetic effects accompanying ATA's use as a therapeutic agent.

## Conclusion and Critical Appraisal

Inhibitors of platelet aggregation currently remain the gold standard for the prevention and treatment of arterial thrombotic events. However, their effectiveness is often limited by bleeding risks and variability in treatment response, highlighting the need for alternative therapeutic strategies. In this review, we have outlined a range of therapeutic agents that target either the function or production of VWF. These agents, along with their mechanisms of action and stages of development, are summarized in Table 1 .

While development of therapeutic VWF inhibitors began as early as the 1980s, most candidates did not progress beyond preclinical or early clinical phases. Several factors may have contributed to this stagnation: limited translational value from animal models to humans, restricted funding, and a predominant research focus on VWF-targeted therapies in the context of VWD rather than thrombotic disorders. Moreover, conventional antiplatelet agents like aspirin, despite their suboptimal efficacy, are inexpensive and widely available, in contrast to the higher production costs and delivery complexity of monoclonal antibodies, nanobodies, and RNA therapeutics.

Nevertheless, recent developments signal a renewed momentum in this field. The approvals of caplacizumab for aTTP and rADAMTS13 for cTTP underscore the feasibility and clinical utility of targeting VWF. Parallel to these advancements, RNA therapeutics have gained significant traction, not only in thrombosis and hemostasis but also across broader therapeutic areas. The approval of siRNA-based drugs nearly every year since 2018, including the recent approval of fitusiran for hemophilia A and B, 129 exemplifies this emerging platform's promise.

Interestingly, the majority of compounds discussed in this review act on the VWF-GPIbα interaction, either by competitively binding the A1 domain of VWF or by blocking the GPIbα receptor on platelets. Given the pivotal role of this axis in shear-dependent platelet adhesion and plug formation, these agents directly interfere with the primary hemostatic function of VWF, highlighting the rationale for targeting this interaction. While this approach is effective in principle, it may also carry a higher risk of bleeding.

An alternative and more upstream strategy involves the reduction of VWF production, as exemplified by siRNA-based approaches currently in early-phase development. In our allele-selective *VWF* silencing approach, we aim to lower elevated VWF levels in arterial thrombosis patients to reduce the risk of residual thrombotic events. This strategy is anticipated to achieve approximately 50% VWF reduction. However, in individuals with extremely elevated baseline VWF levels (e.g., 500 IU/dL), this may not suffice. In such cases, nonselective siRNAs targeting both alleles might offer more profound suppression, though this would require careful dosing to avoid bleeding. Further studies are warranted to define the optimal therapeutic window and determine the precise extent of VWF inhibition needed for thrombosis prevention without compromising hemostasis.

In conclusion, although the therapeutic targeting of VWF remains a promising strategy, particularly in arterial thrombosis and TTP, several clinical and translational challenges remain. Future research should focus on refining these approaches to balance efficacy with safety and to tailor interventions to individual patient profiles.
